# Metabolic Changes After a 24-Week Soccer-Based Adaptation of the Diabetes Prevention Program in Hispanic Males: A One-Arm Pilot Clinical Trial

**DOI:** 10.3389/fspor.2021.757815

**Published:** 2021-11-12

**Authors:** Jennifer K. Frediani, Jianheng Li, Alan Bienvenida, Melinda K. Higgins, Felipe Lobelo

**Affiliations:** ^1^Nell Hodgson Woodruff School of Nursing, Emory University, Atlanta, GA, United States; ^2^Rollins School of Public Health, Emory University, Atlanta, GA, United States

**Keywords:** diabetes, soccer, football, prediabetes, sport, prevention

## Abstract

**Aims:** One third of the U.S. adult population is estimated to have obesity-associated prediabetes. Hispanics have a 50% higher type 2 diabetes death rate compared to non-Hispanic whites, yet low participation in lifestyle change programs, making this subgroup an important target for prevention. Our objective was to determine the feasibility and the effects of an intervention implementing the Center for Disease Control and Prevention National Diabetes Prevention Program (NDPP) plus recreational soccer (RS) in Hispanic men.

**Methods:** Overweight and obese Hispanic men, aged 30–57 years with prediabetes at screening were recruited (*n* = 41). Trained soccer coaches led 30-min facilitated discussion of the NDPP modules after each RS session, with two sessions per week for 12 weeks and once per week for the following 12 weeks. The 1-h RS sessions followed the Football Fitness curriculum. Assessments included body mass index, waist circumference, bioelectrical impedance analysis (InBody 270), blood pressure, glycated hemoglobin (HbA1c), and validated physical fitness tests. Multilevel mixed models assessed the outcomes as a function of time and cohort and incorporated an unstructured covariance structure to examine the changes from baseline to 24 weeks. All analyses were conducted as intent-to-treat using SAS v 9.4.

**Results:** Hispanic males (*n* = 41; mean age 41.7 [0.1] years) were obese at baseline (mean BMI 32.7, standard error of mean [0.7], mean weight 93.9 [2.2] kg). Attendance rate was 65% overall at 12 weeks but differed between cohorts. Five mild injuries occurred over the trial. After 24 weeks of the NDPP+RS intervention, there were significant decreases in systolic and diastolic blood pressure (%change −4.7[SE 2.4]; 95% CI [−11.5, −1.7] and −6.1 [1.7] mmHg; [−9.6, −2.6], respectively), HbA1c (−0.2 [0.1]; [−0.3, −0.1]), Despite significant reductions in weight (−3.8 [0.7]; [−5.2, −2.5]), waist circumference (−6.6 [0.7] cm; [−8.0, −5.1]), body fat % (−1.9 [0.5]; [−2.8, −1.0]), lean body mass was preserved (−0.9 [0.3]; [−1.6, −0.2]).

**Conclusion:** A 24-week soccer-based adaptation of the Diabetes Prevention Program is safe and feasible among middle-aged Latino men.

## Introduction

One third of the US adult population, including 41 million men are estimated to have prediabetes. These individuals are up to 12 times more likely to develop type 2 diabetes (T2DM) than adults with normal glucose tolerance (Centers for Disease Control Prevention, 2017). With an annual attributable mortality of over 80,000, T2DM is the 7th leading cause of death in the US, producing a growing economic burden of over $327 billion. Hispanics/Latinos have a 12.5% T2DM diagnostic rate and have a 50% higher death rate compared to non-Hispanic Whites (Spanakis and Golden, 2013; Centers for Disease Control Prevention, 2017). This combined with the lack of enrollment in the National Diabetes Prevention Program (NDPP) makes this subgroup an important target for prevention efforts.

The NDPP has been successful in reducing the incidence of T2DM through participants' attainment of moderate weight loss and physical activity (PA) goals [Diabetes Prevention Program (DPP) Research Group, 2002]. Cultural adaptation and implementation studies show positive results in translating the NDPP by leveraging community-based resources (Ali et al., 2012; Tabak et al., 2015). Although some studies have adapted the NDPP to be culturally appropriate to high-risk groups such as Hispanics, these have predominantly targeted females (Merriam et al., 2009; Ruggiero et al., 2011). Males, in general, are harder to engage in screening and disease prevention programs, including lifestyle interventions (Hunt et al., 2013). Although more than 250,000 adults in the US have completed the NDPP, men comprise <20% of enrolled adults and Hispanic/Latinos are the racial/ethnic group with the lowest participation (10%) (Gruss, 2018).

As the most widely played team sport in the world, soccer/football has massive potential as a conduit for successful delivery of lifestyle interventions (Barriguete Melendez et al., 2014; Bellissimo et al., 2018). Already, research groups in Europe and South America have delivered recreational soccer (RS) based programming to a wide range of populations (Hunt et al., 2014; van Nassau et al., 2016). Compared to enrollment in the NDPP, soccer-related health interventions are shown to be more successful at recruiting and retaining men in traditional disease prevention programs compared to typical diet and physical activity programs that do not involve soccer (Hunt et al., 2013).

With growing potential, RS interventions have been shown to be an effective method of engaging hard to reach populations and reducing cardiometabolic risk factors associated with diabetes (Krustrup et al., 2010). Hispanic men, in particular, do not attend NDPP programs nationwide, making up only 10% of all participants between 2012 and 2016 (Ely et al., 2017). The NDPP is a classroom-based program that rarely includes physical activity sessions, with some programs also run online. The original diabetes prevention program study emphasized physical activity and found it to be a major driver of weight loss (Diabetes Prevention Program Research Group, 2002).

Previous research focused on promotion and adaptation of the NDPP in Hispanic populations have identified language barriers, low levels of health literacy, economic constraints, lack of transportation, and fear of participating in research as barriers to recruitment. In order to mitigate these factors, several research studies have identified the importance of community engagement, cultural and linguistic tailoring of the NDPP, and the employment of culturally competent lifestyle coaches (Ockene et al., 2011; Vincent et al., 2013; Ritchie et al., 2018). Overall, RS programming can be tailored to incorporate all of these beneficial factors to increase recruitment and improve retention of Hispanic men in the NDPP.

Thus, given its fun, social, and competitive nature, our research team adapted and implemented a RS-based version of the NDPP in Hispanic men living in Atlanta, Georgia. Our objective was to determine feasibility and safety, and the cardiometabolic effects of this lifestyle intervention (adapted NDPP plus recreational soccer). We hypothesized that the participants would enjoy the program and improve all cardiometabolic markers. To our knowledge, no research exists which attempts to combine the traditional NDPP program, with an exercise component such as RS, for Latino men.

## Subjects, Materials, and Methods

### Participants

#### Study Inclusion and Exclusion Criteria

To participate in the study, the participants had to be: (1) Hispanic/Latino men aged 35–55 years; (2) Body mass index (BMI) ≥ 25 kg/m^2^; (3) elevated risk for diabetes, operationalized as a score ≥9 in the Center for Disease Control and Prevention (CDC) pre-diabetes risk calculator (Centers for Disease Control Prevention, 2016); (4) not engaged in regular soccer practice or other physical activity (PA) or lifestyle intervention program in the last year; (5) ability to read in English or Spanish and to provide inform consent. Participants were excluded if: (1) they reported a type 2 diabetes diagnosis or medication (oral or injected); (2) BMI ≥ 40; (3) resting blood pressure ≥170/100 at screening; (4) any mobility issues or contraindications for high intensity interval training (HIIT) PA program (Riebe et al., 2015). All participants were informed of risks and provided informed consent in the language of their choice. The study was carried out in accordance with the guidelines set by the Institutional Review Board at Emory University (IRB#00100342).

### Study Design

**Figure 1:**
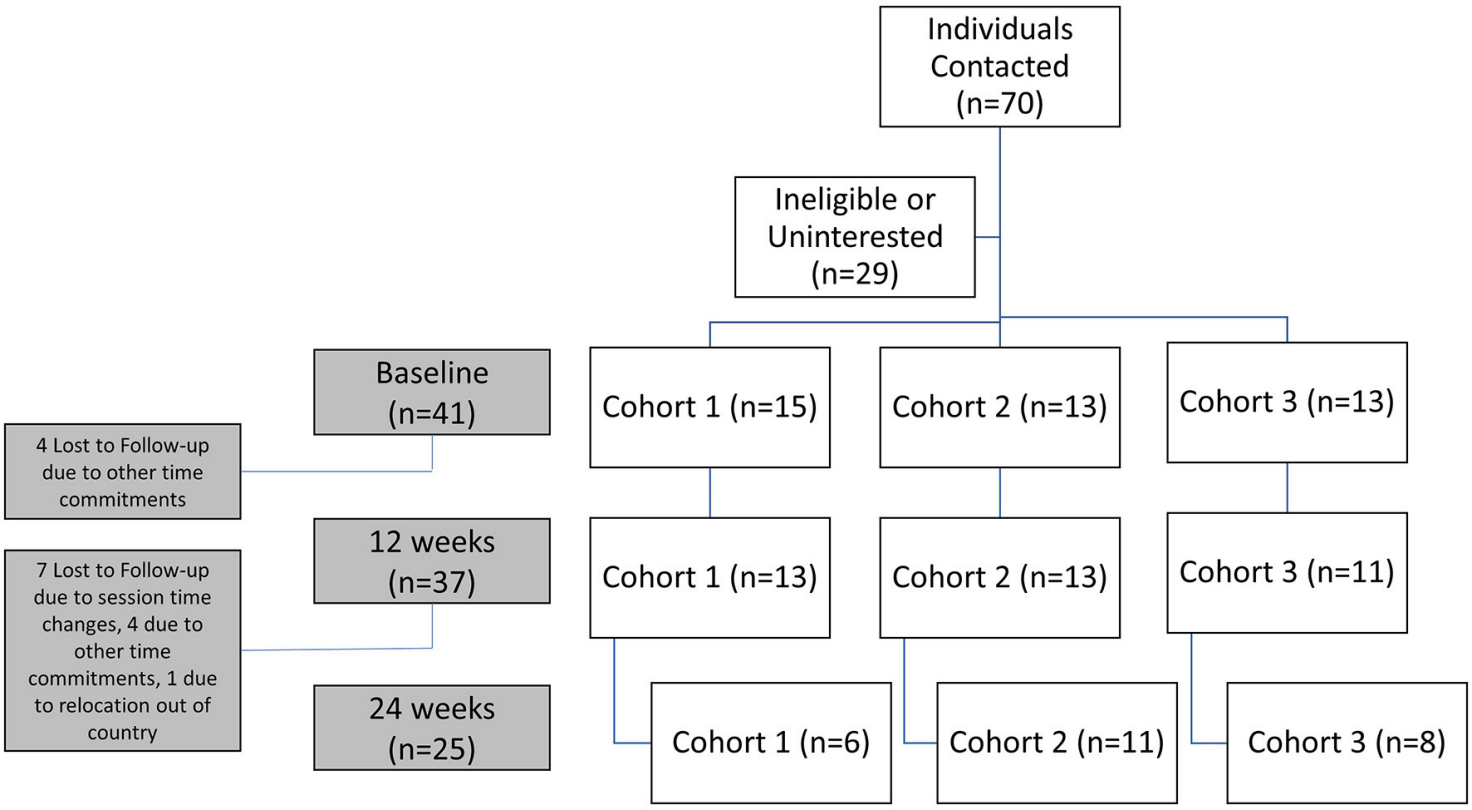
Recruitment flow chart from individuals contacted to final cohorts at 24 weeks.

This feasibility pilot study was a one-arm 24-week clinical trial. A total of 41 participants were recruited between April and July 2018 (ClinicalTrials.gov Identifier: NCT03595384) (Soccer-based Adaptation of the Diabetes Prevention Program–Full Text View–ClinicalTrials.gov, 2018). Participants were recruited from local Atlanta area Latino organizations using online (social media, websites, email listservs) and print (study recruitment fliers) advertisements methods. Recruitment flow chart from recruitment to 24-week measurements is found in Figure 1. Interested participants were provided an online survey to ascertain eligibility (CDC pre-diabetes screening, demographic questions, and PA habits) and those meeting inclusion criteria were invited to an initial session at a local soccer field for informed consent and confirmatory baseline assessments including BMI, blood pressure, glycated hemoglobin (HbA1c) and exercise pre-participation risk screening. Prior to testing, subjects were asked to refrain from physical activity for 48 h prior to test day, refrain from alcohol 24 h prior to testing, and refrain from caffeinated drinks, smoking and other stimulants 1 h prior to testing. Siemens DCA Vantage HbA1c point-of-care devices (Erlangen, Germany) were used for diabetes screening (Siemens Healthineers, 2017). A cut off point of 6.5% was used as a putative definition of diabetes and these individuals were referred to a local clinic for confirmatory testing and appropriate management. Both systolic and diastolic blood pressure were measured while seated using a calibrated electronic blood pressure sphygmomanometer (Omron, Kyoto, Japan) prior to any activity and after 5 min of rest. Participants meeting inclusion and exclusion criteria were invited to participate in the study.

### Outcomes

The primary outcome of this study was feasibility. The secondary outcomes were body composition and weight measured by bioelectrical impedance analysis, waist circumference, HbA1c, blood pressure, and dietary recalls. Secondary outcomes of physical assessments including vertical jump, agility, predicted VO2 max, push-ups and sit-ups, resilience, and behavior regulation in exercise were all published elsewhere (Frediani et al., 2020). Assessments were conducted at baseline before the first soccer session, after the 12-week initial soccer conditioning phase, and at 24-weeks after the soccer league (maintenance phase of the program). Participants were all briefed on the various measurements prior to the tests and all study staff were trained and demonstrated ability to perform measurements.

### Anthropometrics

Body weight, body fat percentage and lean body mass were measured in light exercise clothing and not wearing shoes, using bioelectrical impedance analysis (InBody 270, Anaheim, CA, USA). Height was measured using a stadiometer (Seca^®^ 213, Hamburg, Germany). Waist circumference was measured in centimeters using a tape measure (Ohaus^®^ 8004-MA, Parsippany, NJ, USA) at the smallest circumference around the torso between the bottom of the xiphisternum and the top of the iliac crests. Each measurement was taken twice to account for accuracy. If the first two measurements were >2 cm then a third measurement was taken and the closest two were averaged (Centers for Disease Control Prevention, 2007).

### Dietary Assessment

At each measurement point, one 24-h dietary recall was completed and a second one was done by phone within 2 weeks in English or Spanish according to participant's preference. The validated multi-pass method was used by trained staff (Steinfeldt et al., 2013). Nutrition Database System for Research software (NDSR) (Nutrition Coordinating Center, University of Minnesota, Minneapolis, MN, USA) was used to analyze all dietary recalls.

### Program Delivery Personnel

Three bilingual coaches (Fédération Internationale de Football Association (FIFA) or US Soccer certificate) were recruited and trained on intervention procedures. They all completed the “Football is Medicine” training course (6 h theory, 2 h practice) and received a copy of the Coaches “Football Fitness Manual” with drills and exercises (Krustrup et al., 2018). They also received training on safety precautions according to the American College of Sports Medicine Exercise Pre-participation Guidelines and obtained a certification in cardio-pulmonary resuscitation (Riebe et al., 2015). Furthermore, they were also trained by a certified NDPP lifestyle coach on how to deliver and facilitate the NDPP Prevent T2 curriculum.

### Intervention

After the baseline assessments, participants were asked to attend group soccer practice twice a week for a 12-week period designed to replicate the concept of “pre-season conditioning.” Three groups of 13–15 participants were assembled, assigned a coach and session scheduling was tailored to the group's preference based on field availability and location convenience for participants. Sessions were held in the evenings (typically 7 p.m. Friday and Sunday) allowing at least 48 h rest for recovery and to maximize physiologic adaptations. Each RS conditioning session included an evidence-based 15-min dynamic warm-up (FIFA 11+) (Federation Internationale de Football Association, 1994–2021), 20 min of soccer-specific drills with and without the ball and 20 min recreational short-sided soccer games 5v5 up to 7v7 format depending on session attendance. During this 12-week period, participants completed the NDPP core curriculum modules (one/week). At the end of the soccer session and while hydrating and cooling down, participants sat in a group to discuss the NDPP modules facilitated by their coach, during the last 30 min of each 85-min study session. Sessions were held in parallel for the three cohorts on a full-size artificial turf soccer field. Participants were encouraged to bring family and/or friends as spectators for additional social support.

A closed and encrypted chat (WhatsApp platform) was created for each cohort and for the full group to facilitate peer-support, communication, and session attendance (i.e., schedule changes). Coaches provided attendance encouragement weekly, and participants also used it to post encouraging messages and provide support (i.e., picture examples of their dietary choices, activity levels outside of soccer sessions, etc.). Coaches recorded session attendance, weekly weights and rates of perceived exertion and reported any injuries or adverse events at the end of each session.

### Data Analysis and Sample Size

All analyses were conducted as intent-to-treat. Multilevel mixed models with unstructured heterogeneous covariance structures, were used to analyze outcomes at each time point. Linear mixed models were used to assess cohort by week interaction, *p*-value ≤ 0.05 indicates the changes from baseline to 24 weeks were different across the three cohorts. We assumed the data was missing at random due to the most common reason for loss to follow up was time management, and the maximum likelihood estimation procedure was used to handle missing data (19.5% missing). For each outcome measure, the model assessed the outcome as a function of time (baseline, 12 and 24-weeks) and adjusted for assigned cohort. Given the exploratory nature of this study no adjustment for the multiple comparisons was made. All dependent variables were modeled using fixed effects. Least square means adjusted for cohort effect were reported for each outcome. Data are expressed as means (standard error of the mean [SEM]). Hypotheses were two sided and tested at a 0.05 significance level, 95% confidence intervals were reported. Analyses were conducted using SAS 9.4 (SAS Institute, Cary, North Carolina, USA). We calculated that 40 participants would provide at least 86% power to detect a mean difference in PA as the primary outcome (100 min/week; SD 200 min) and over 90% for detecting significant changes in weight (1.45 kg; SD 0.18) and VO_2_max (3.93 ml/kg/min; SD 0.5) from baseline to 24 weeks using a two-sample *t*-test (Frediani et al., 2020).

## Results

Baseline demographics are shown in Table 1. The mean age of the 41 participants was 41.9 (SD = 6.2) years old. Most of the participants were married (83%), were employed (93%), and 43.6% had a college or technical school degree. A little more than half (51%) had a family history of diabetes (14.6% had a sibling and 43.9% a parent with diabetes).

**Table 1:** Demographics.

**Characteristics**	**N (%)**
Age, mean (SD), range	41.9 (6.2), 32–57
Family history of diabetes	21 (51.2)
Have a sibling with diabetes	6 (14.6)
Have a parent with diabetes	18 (43.9)
Country of origin	
Mexico	16 (39.0)
Venezuela	4 (9.8)
Ecuador	3 (7.3)
Guatemala	4 (9.76)
Colombia	7 (17.1)
Other	7 (17.1)
Marital status	
Single	2 (4.9)
Married	34 (82.9)
Domestic partner	2 (4.9)
Divorced	2 (4.9)
Widowed	1 (2.4)
Education	
Primary school	3 (7.3)
Some high school	4 (9.8)
High school	10 (24.4)
College degree	18 (43.9)
Tech school	4 (9.8)
Employment	
Work for pay in a business or job	37 (90.2)
Look for work	1 (2.4)
Did not have a job or business and was not looking for work	2 (4.9)
Smoking	
Current smoker	2 (4.9)
Former smoker	3 (7.3)
Never smoke	11 (26.8)
Missing	25 (61.0)
Medical conditions	
Obesity	30 (73.2)
Hypertension	37 (90.2)
Prediabetes or diabetes	25 (61.0)
Medications	
Anti-hypertensive	12 (29.3)
Anti-diabetic	2 (4.9)
Anti-hyperlipidemic	3 (7.3)

### Feasibility and Adherence

**Figure 2:**
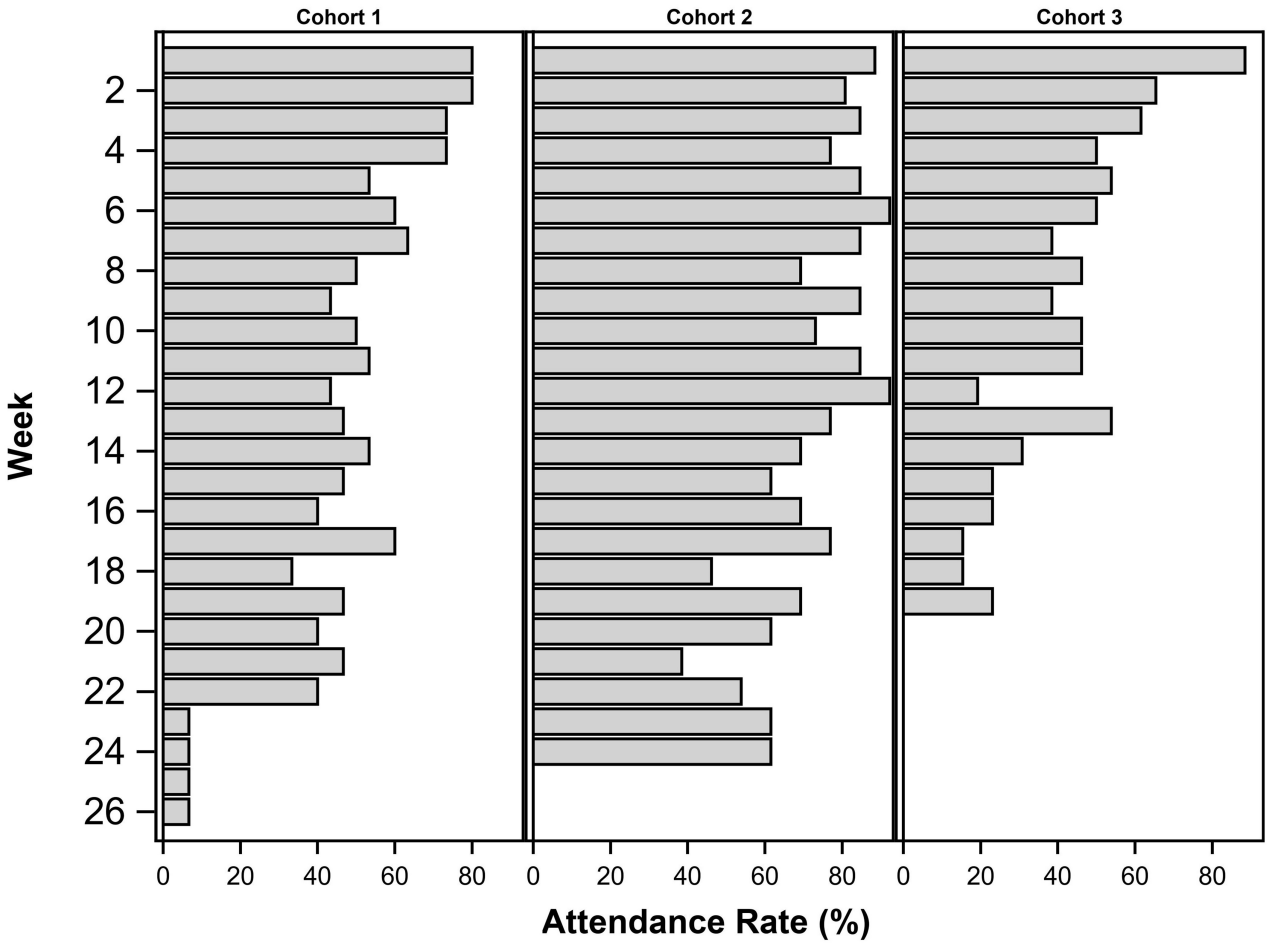
Mean attendance rates (%) by cohort and overall between Baseline, 12 and 24 weeks.

Attendance differed between the three cohorts and between the first 12 weeks and the second 12 weeks of the program when the frequency of soccer sessions decreased. Figure 2 shows the average attendance rate by cohort and study phase. Cohort 2 had the highest attendance for both study phases and all cohorts decreased after the first 12 weeks. There was a total of 32 planned sessions and the average attendance was 20.4 or 65%. Most common reasons for missing sessions were time management and travel. Participant satisfaction was assessed at the end of the 24-week program. The questionnaire included questions about the coach, both during soccer sessions and as a facilitator of the NDPP sessions, use of the Garmin device, group discussions and handouts, convenience of the sessions time and location, and the program as a whole. All participants reported high overall satisfaction with the lifestyle program with an average of 9.9 out of 10. There were five mild injuries reported among the 41 participants. These included hamstring or calf strains and Achilles' tendinitis flares. All injuries resolved within 2–3 weeks and no other adverse events occurred. Rate of perceived exertion were averaged by cohort for the first three sessions and the last three sessions. They ranged between 6.6 and 7.7 (modified Borg scale 1–10) (Borg et al., 1985) at the beginning of the intervention and 3.4–5.7 for the end of the intervention. Intensity and duration during soccer sessions have already been reported (Frediani et al., 2020). Briefly, we utilized Playertek devices and the accompanying Catapult software to measure soccer specific metrics including session duration, overall distance, sprint distance and estimated energy expenditure (PLAYERTEK | Affordable Athlete Tracking System | Catapult Sports, n.d.). All metrics improved at the 12-week time point and then decreased at the 24-week time point (Frediani et al., 2020).

### Body Composition

**Figure 3:**
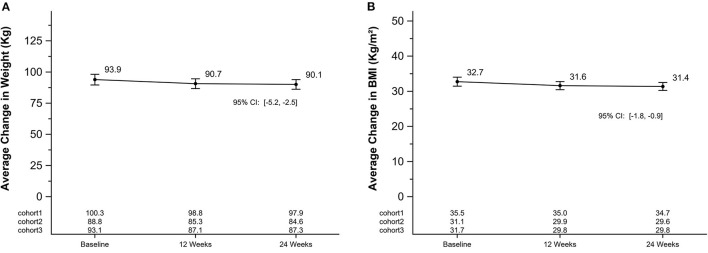
Average change in weight and BMI in a 24-week soccer-based diabetes prevention intervention in Hispanic men. **(A)** Changes in mean weight (kg) at baseline, 12 and 24 weeks. P for cohort by week interaction: 0.02. **(B)** Changes in mean BMI (kg/m^2^) at baseline, 12 and 24 weeks. P for cohort by week interaction: 0.13. BMI, body mass index.

**Figure 4:**
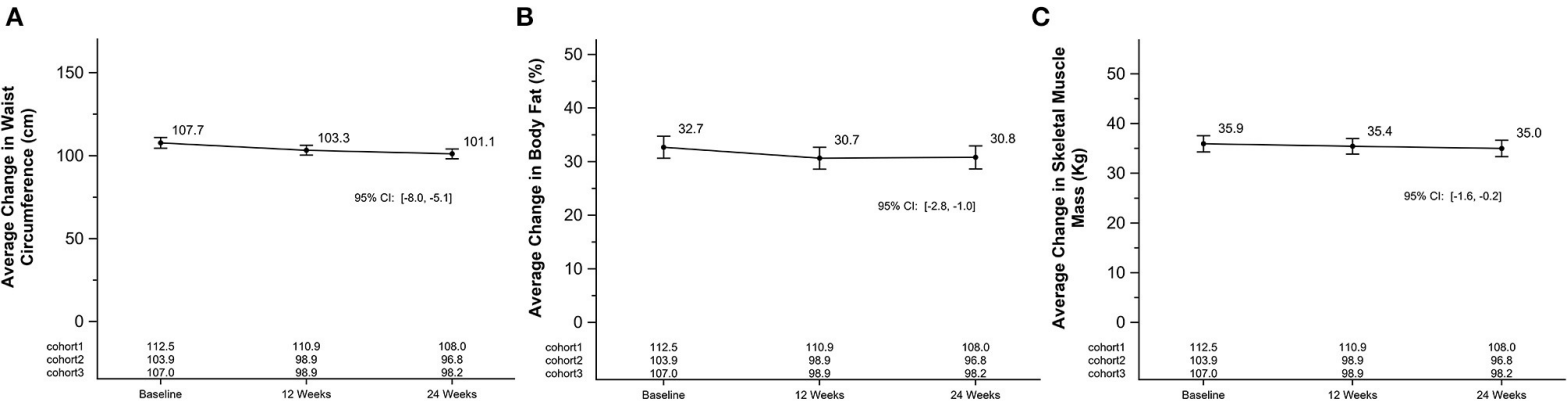
Average change in waist circumference, body fat percentage, and skeletal muscle mass in a 24-week soccer-based diabetes prevention intervention in Hispanic men. **(A)** Changes in mean waist circumference (cm) at baseline, 12 and 24 weeks. P for cohort by week interaction: 0.03. **(B)** Changes in mean body fat (%) at baseline, 12 and 24 weeks. P for cohort by week interaction: 0.03. **(C)** Changes in mean skeletal muscle mass (kg) at baseline, 12 and 24 weeks. P for cohort by week interaction: 0.03.

On average, weight, BMI, waist circumference and body fat percentage decreased by 3.3 Kg (SEM = 0.7; 95% CI [−4.6, −1.9]), 1.1 kg/m^2^ (SEM = 0.2; [−1.6, −0.7]), 4.4 cm (SEM = 0.8; [−6.0, −2.7]), 2.0% (SEM = 0.3; [−2.7, −1.4]) after 12 weeks, respectively. Differences between baseline and 24-weeks also showed significant declines for weight, BMI, waist circumference, and body fat percentage by 3.8 kg (SEM = 0.7; 95% CI [−5.2, −2.5]), 1.4 kg/m^2^ (SEM = 0.2; [−1.8, −0.9]), 6.6 cm (SEM = 0.7; [−8.0, −0.9]), 1.9% (SEM = 0.5; [−2.8, −1.0]), respectively. Skeletal muscle mass also declined between baseline and 24-weeks by 0.9 kg (SEM = 0.3; [−1.6, −0.2]). Figure 3 shows mean changes for weight and BMI and Figure 4 shows mean changes for waist circumference, body fat percentage and skeletal muscle mass at each measurement point. There was a cohort by week interaction for body weight (*p*-value = 0.05), waist circumference (*p*-value = 0.03), body fat percentage (*p*-value = 0.03), and skeletal muscle mass (*p*-value = 0.03), but not for BMI. Each cohort mean is listed by timepoint on Figures 3, 4 for each panel.

### Cardiometabolic Biomarkers

**Figure 5:**
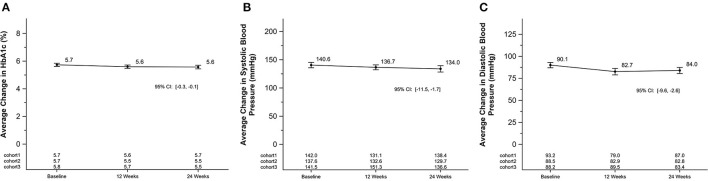
Average change in HbA1c and blood pressure in a 24-week soccer-based diabetes prevention intervention in Hispanic men. **(A)** Changes in mean HbA1c (%) at baseline, 12 and 24 weeks. P for cohort by week interaction: 0.23. **(B)** Changes in mean systolic blood pressure (mmHg) at baseline, 12 and 24 weeks. P for cohort by week interaction for systolic: <0.01. **(C)** Changes in mean diastolic blood pressure (mmHg) at baseline, 12 and 24 weeks. P for cohort by week interaction for diastolic: <0.01. HbA1c, glycated hemoglobin; mmHg, millimeters of mercury.

There were significant reductions in diastolic blood pressure after 12-weeks (−7.4 [2.2] mmHg; [−11.8, −2.9]). Reductions in systolic blood pressure and HbA1c were found at 12-weeks although not as profound (−3.9 mmHg; SEM = 2.6; [−9.2, 1.4]) and −1 mmol/mol (−0.1%); SEM = 1 mmol/mol (0.1%); [−3,0]) (Figure 5). After 24-weeks, all cardiometabolic markers were significantly reduced (systolic blood pressure −6.6 mmHg; SEM = 2.4; [−11.5, −1.7], diastolic blood pressure −6.1 mmHg; SEM = 1.7; [−9.6, −2.6], and HbA1c (−2 mmol/mol (−0.2%); SEM = 1 mmol/mol 0.1; [−3, −1]) (Figure 5). All changes in cardiometabolic markers were clinically significant. The change in HbA1c was minimal in this prediabetes population due to the baseline values averaging <42 mmol/mol (6%). There was a cohort by week interaction for blood pressure (systolic *p*-value < 0.01; diastolic *p*-value < 0.01), but not for HbA1c. Each cohort mean is listed by timepoint on Figure 5 for each panel.

### Nutrition

**Figure 6:**
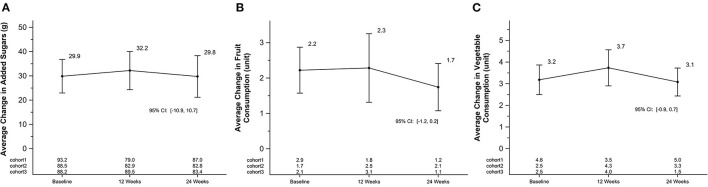
Average change in added sugars, fruit, and vegetable consumption in a 24-week soccer-based diabetes prevention intervention in Hispanic men. **(A)** Changes in mean added sugar intake (g) at baseline, 12 and 24 weeks. P for cohort by week interaction: <0.01. **(B)** Changes in mean fruit consumption in servings (USDA Dietary Guidelines) at baseline, 12 and 24 weeks. P for cohort by week interaction: 0.03. **(C)** Changes in mean vegetable consumption in servings (USDA Dietary Guidelines) at baseline, 12 and 24 weeks. P for cohort by week interaction: <0.01. USDA, United States Department of Agriculture.

Fruit and vegetable servings and added sugar were determined by NDSR as stated above. There were no significant changes in fruit and vegetable consumption over the course of the study period. Differences shown in Figure 6. Added sugar (grams) was also not significantly different between baseline, 12 and 24 weeks (Figure 6). Total energy and total fat intake did not statistically differ over time from baseline (Total energy–Baseline: 6740 kj (314) 2992–10,996; 12 weeks: 14%; 24 weeks: −7%; Total Fat—Baseline: 63 g (4) 18–123 g; 12 weeks: 14% 24 weeks: −11%). There was a cohort by week interaction for added sugar intake (*p*-value < 0.01), mean fruit consumption (*p*-value = 0.03), and vegetable consumption (*p*-value < 0.01). Each cohort mean is listed by timepoint on Figure 6 for each panel.

## Discussion

This one-arm pilot clinical trial to deliver a soccer-based adaptation of the NDPP in Hispanic men with prediabetes was safe and feasible to implement. The study resulted in improved BMI, waist circumference, body fat %, HbA1c, and blood pressure over the course of a 24-week intervention. Our participants expressed high satisfaction with the program and sustained minimal mild injuries during the trial.

The adherence rate was 65% or 20 out of 32 planned sessions. The participant-level evaluation of the CDC's National Diabetes Prevention Program reported an adherence rate of 48% with an average duration of 16 weeks (16% of participants). This pilot trial was well above the national average (Ely et al., 2017). This indicates that the addition of recreational soccer increased engagement when compared to the traditional version of the NDPP. Our attendance was lower after the midpoint where the frequency of soccer sessions decreased from twice per week to once per week. The participants in cohort 1 did not respond well to the change and fewer attended regularly afterwards. Secondly, the project ended the third week of December causing the third cohort's lower attendance toward the end of their 24-week period. We found significant interactions between cohorts over time for most biological measures. We suspect the differences between both the participants' personalities and the coaches' style perhaps influenced the study. In addition, individual discussions between participants were not recorded but may have included additional information that differed between groups particularly pertaining to nutrition. All NDPP modules were scripted, and all coaches were trained to follow the Football Fitness Curriculum for all soccer sessions. This study's recruitment method differed from others because the participants were separated into smaller groups to allow more playing time and to facilitate discussion of NDPP modules in smaller groups. All other RS intervention studies are based outside of the US and did not include an educational component and poorly reported compliance overall. Luo et al. found on average for short trials (~12 week duration) attendance rates were ~78% and for longer trials (24–32 weeks) the average was 66% when compared to a resistance training control group (Luo et al., 2018).

On average, body composition and anthropometric parameters significantly improved among participants in this lifestyle intervention study. Despite the lack of control group, our findings were similar to changes seen in previous recreational soccer studies when compared to non-exercise controls. Mohr et al. saw a higher decrease in body fat percentage (3%) as measured by dual energy x-ray absorptiometry after 16-weeks of recreational soccer plus dietary intervention in slightly older men than the present study (Mohr et al., 2019). These findings were further corroborated in three other studies. Andersen et al. found a total fat mass reduction of 1.7 kg in middle aged men with type 2 diabetes (Andersen et al., 2014). Another study in adults with type 2 diabetes saw a reduction in fat mass (−3.4 kg) before and after an intervention of soccer plus diet intervention after 12 weeks (de Sousa et al., 2014). Randers et al. found a 3.2 kg reduction in 30–40 year old untrained men after 12 weeks (Randers et al., 2010). Similarly, a reduction in waist circumference (4 cm) was seen after 16 weeks, where we saw the same decline at 12 weeks (Mohr et al., 2019).

Lean body mass declined by −3% from baseline in our study and this was also seen in another study of adults with type 2 diabetes (de Sousa et al., 2014). Traditional lifestyle-based weight loss interventions among overweight/obese participants result in a comparatively larger (4–30%) muscle mass loss from baseline (Michalczyk et al., 2020; Turicchi et al., 2020). Muscle mass loss can also be associated with weight regain. Our findings could be the result of dietary changes in combination with the increased physical activity. For example, through a decrease in protein intake, which was not examined in this study. However, the combination of calorie restriction and exercise should attenuate the loss of lean mass (Weiss et al., 2017). The nature of the RS intervention approach, which combines aerobic, HIIT and resistance training exercise in each session) may help mitigate to some extent the typical decline in muscle mass seen in traditional lifestyle intervention trials.

We found clinically significant improvements in blood pressure in our study, decreased from a hypertensive mean toward the normal range after 24-weeks. A clinically meaningful decline in blood pressure can be defined as −2 mmHg (Turnbull and Blood Pressure Lowering Treatment Trialists' Collaboration, 2003). Most exercise interventions involving recreational soccer training have seen similar results (Milanović et al., 2019). Our study falls in the median of blood pressure reduction. Flotom et al. saw a −3.5 mmHg systolic drop and −2.3 mmHg diastolic drop in 18 weeks (Fløtum et al., 2016) and others reached a larger reduction of 10.8 mmHg systolic and 7.3 mmHg diastolic in 16 weeks (Mohr et al., 2019). These two studies were in non-hypertensive and hypertensive samples, respectively.

Modest improvements in HbA1c were seen during the 24-week intervention, with a larger reduction within the first 12 weeks. The measurement sessions, as well as the RS sessions, were all conducted in the evenings to facilitate participation given the participants work schedules, therefore fasting glucose measurements were not possible. Most other recreational soccer studies measured fasting blood glucose instead of HbA1c (Andersen et al., 2014; Mohr et al., 2019). However, de Sousa et al. measured HbA1c in a group of adults with T2DM and found a similar reduction between their soccer plus diet group and the diet only group post intervention after 12 weeks (de Sousa et al., 2014). Similarly, fasting blood glucose was lowered in all groups post-intervention when recreational soccer plus dietary intervention was compared to dietary intervention alone, with a larger reduction in the soccer group in a study of older adults (Mohr et al., 2019).

We found that RS sessions provide both aerobic and anaerobic physiological benefits. During an RS session, participants can engage in more than 100 high-intensity functional movements such as springs, jumps, dribbles, and abrupt changes in direction and speed, over a 60-min session. These HIIT intervals result in a beneficial cardiovascular workout, with participant heart rates averaging 80% of maximal exertion. As evidenced by our study and others, RS participants exhibit a reduction in insulin resistance, decrease in chronic inflammation and arterial stiffness and an overall improvement in non-communicable diseases (de Sousa et al., 2014; Bangsbo et al., 2015; Sarmento et al., 2020). Not only do RS sessions result in cardiometabolic risk reduction, they are also risk adverse (Rognmo et al., 2004; Jung et al., 2015; Ramos et al., 2015) and increase social capital of participants (Blatter and Dvorak, 2014).

Overall nutrition did not change over time in this study. The nutrition intervention portion of this study was embedded in the adaptation of the NDPP, therefore this study was analyzed as a comprehensive lifestyle intervention. We selected several key messages from the program from the Spanish version of the PreventT2 curriculum (Curricula Handouts | NDPP | Diabetes | CDC, 2019). Those pertaining to nutrition were Healthy Eating, Tip the Calorie Balance, four Keys to Healthy Eating Out, Have Healthy Food You Enjoy, More about Carbs, and Handling Holidays, Vacation and Special Events. Participants completed the NDPP's food trackers and often discussed their meals on the closed WhatsApp groups. However, these methods did not lead to a noticeable change in macronutrient distribution or fruit and vegetable servings or added sugar intake as measured via dietary recalls. We can only speculate why this may have occurred. One reason could be delivery of the curriculum modules. Although, all soccer coaches were trained on NDPP facilitation and were given scripts for each education session, the degree of nutrition recommendations they were able to provide was variable. However, the NDPP was designed to be delivered using a facilitated discussion approach not necessarily with a nutrition lecture. It also does not “adhere” to a specific diet or nutrition guidance beyond general recommendations to reduce carbohydrates and fat consumption. Another reason could be the influence of the participants' families and the reliance of popular media nutrition information and current fad diets at the time. In classroom-based NDPP programs, Hispanics, especially men are poorly represented and have low attendance (Ritchie et al., 2018, 2019). Regardless, follow up studies may evaluate the impact of specified nutrition education sessions conducted by a registered dietitian rather than relying on facilitation of the defined nutrition modules.

This study was a single arm, unblinded clinical trial aimed to determine the feasibility and explore efficacy signals of a novel delivery method of diabetes prevention education in Hispanic men. Our strengths lie in our culturally sensitive approach and our ability to conduct this intervention in Spanish. We also used methods to increase positive group dynamics within the cohorts. We are limited in our generalizability as we focused on Hispanic middle-aged men in this pilot. We also did not have a control group to compare our results in a usual care population. We did not evaluate participant satisfaction about the Prevent T2 curriculum, given that cultural adaptations of the NDPP curriculum for Spanish-speaking populations had already been performed.

In summary, a soccer-based adaptation of the National Diabetes Prevention Program delivered in Spanish by bilingual soccer coaches to a group of Hispanic men was both feasible and safe over a 24-week period. Future studies are needed to further evaluate the intervention and directly assess the efficacy of this promising RS-based NDDP adaptation in comparison with the traditional “sit and learn” NDPP delivery method (online and/or face-to-face) as well as potential for scale-up, cost-utility, and translation to other age, gender, racial sub-groups and geographies.

## Data Availability Statement

The raw data supporting the conclusions of this article will be made available by the authors, without undue reservation.

## Ethics Statement

The studies involving human participants were reviewed and approved by Institutional Review Board at Emory University (IRB#00100342). The patients/participants provided their written informed consent to participate in this study.

## Author Contributions

All authors made substantial contributions to conception and design, acquisition of data or analysis, interpretation of data, drafting the article or revising it critically for important intellectual content, and provided final approval of the version to be published.

## Funding

This work was supported in part by grant P30DK111024 from the Georgia Center for Diabetes Translation Research funded by the National Institute of Diabetes and Digestive and Kidney Diseases.

## Conflict of Interest

The authors declare that the research was conducted in the absence of any commercial or financial relationships that could be construed as a potential conflict of interest.

## Publisher's Note

All claims expressed in this article are solely those of the authors and do not necessarily represent those of their affiliated organizations, or those of the publisher, the editors and the reviewers. Any product that may be evaluated in this article, or claim that may be made by its manufacturer, is not guaranteed or endorsed by the publisher.

## Abbreviations

BMI: Body mass index
CDC: Centers for Disease Control and Prevention
FIFA: Fédération Internationale de Football Association
HbA1c: glycosylated hemoglobin
HIIT: high intensity interval training
NDPP: National Diabetes Prevention Program
NDSR: Nutrient Database System for Research
PA: physical activity
RS: recreational soccer
SEM: estimated standard error of the mean
US: United States.
